# Comparison of the curative effect and prognosis of stereotactic drainage and conservative treatment for moderate and small basal ganglia haemorrhage

**DOI:** 10.1186/s12883-021-02293-7

**Published:** 2021-07-06

**Authors:** Xin Huang, Lai Jiang, Shaojun Chen, Gang Li, Wanxi Pan, Lei Peng, Ziwei Yan

**Affiliations:** 1 Department of Neurosurgery, The People’s Hospital of China Three Gorges University, Yichang, China; 2 Department of Ultrasound Diagnostics, The People’s Hospital of China Three Gorges University, Yichang, 443000 China

**Keywords:** Stereotactic drainage, Moderate to small basal ganglia haemorrhage, Complications

## Abstract

**Background:**

Minimally invasive surgery has achieved good results in the treatment of cerebral haemorrhage.However, no large-scale clinical study has demonstrated that surgical treatment of cerebral haemorrhages less than 30 ml can improve the curative effect. Our study explored the efficacy and complication of stereotactic drainage based on the amount of cerebral hemorrhage (15-30 ml) in hypertensive basal ganglia.

**Method:**

Sixty patients with hypertensive basal ganglia haemorrhages were divided into a control group and an experimental group with 30 patients in each group. Patients in the control group were treated conservatively. In contrast, those in the experimental group received stereotactic drainage, and urokinase was injected into the haematoma cavity after the operation. The haematoma volume at admission and 1, 3, 7 and 30 days after treatment and National Institute of Health stroke scale(NIHSS) score data were recorded. Complications after treatment in the two groups of data were compared and analysed.

**Result:**

No significant differences in age, sex, time of treatment after onset, admission blood pressure, admission haematoma volume or admission NIHSS score were noted between these two groups (*P* > 0.05). After treatment, significant differences in haematoma volume were noted between the two groups on the 1st, 3rd, 7th and 30th days after treatment (*P* < 0.05). The amount of hematoma of patients in the experimental group was significantly reduced compared with that in the control group, and the NIHSS scores were significantly different on the 3rd, 7th and 30th days after treatment. The neurological deficit scores of patients in the experimental group were significantly reduced compared with those in the control group, and the incidence of pulmonary infection and venous thrombosis in the lower limbs of patients in the experimental group were significantly reduced (*P* < 0.05). ROC curve analysis showed that stereotactic drainage affected the early neurological function of patients with small and medium basal ganglia haemorrhages.

**Conclusion:**

For patients with small and medium basal ganglia haemorrhages, stereotactic drainage can be used due to the faster drainage speed of haematomas after operation, which is beneficial to the recovery of neurological function and reduce complications.

## Introduction

Hypertensive cerebral haemorrhage is a common disease in neurosurgery that mostly occurs in middle-aged and elderly people with high disability and mortality rates, and the incidence rate gradually increases with the ageing of the population. Hypertensive cerebral haemorrhage accounts for 21 ~ 48% of all stroke patients, among which basal nucleus haemorrhage is the most common symptom, representing approximately 50% of cerebral haemorrhage patients [1, 2]. Patients often have symptoms, such as hemianopia, hemiplegia and hemiparesthesia, because the conduction bundle in the basal ganglia is damaged [3]. If this condition not treated in time to relieve compression and protect nerve tissue, it will easily lead to limb nerve dysfunction, resulting in sequelae and affecting the prognosis of patients. The cerebral haemorrhage treatment methods are classified into medically conservative treatment and surgical treatment [4]. Surgical methods mainly include craniotomy haematoma removal, endoscopic surgery, minimally invasive drilling and drainage [5]. Given the clinical application of multimodal navigation and minimally invasive surgery in medical imaging, it is generally believed that minimally invasive surgery can be performed for patients with bleeding volume > 15 ml. Minimally invasive surgery such as endoscopic surgery and stereotactic drainage, has achieved good results in the treatment of cerebral haemorrhage [6]. For hypertensive intracerebral haemorrhage with a haematoma volume of 20 ~ 30 ml, minimally invasive surgery can obviously speed up the clearance time of hematoma compared with conservative medical treatment [7]. However, no large-scale clinical study has demonstrated that surgical treatment of cerebral haemorrhages less than 30 ml can improve the prognosis. The objective of this study is to clarify the efficacy and complication of stereotactic drilling and drainage and conservative treatment in patients with small- and medium-sized basal ganglia haemorrhages and to provide a basis for the clinical treatment of hypertensive small- and medium-sized basal ganglia haemorrhages [8]. The report is as follows.

## Date and methods

### Research objects

From June 2017 to June 2020, 60 patients with hypertensive intracerebral haemorrhage admitted to the Department of Neurosurgery in our hospital were selected as the research subjects. The random number table method was adopted. In all patients, haemorrhages were located in the basal ganglia, and were randomly divided into an experimental group and a control group with 30 patients in each group. All the studies were approved by the hospital ethics committee, Patients and their families and voluntarily signed relevant informed consent forms from the hospital. Inclusion criteria: (1) According to the "Guidelines for Diagnosis and Treatment of Cerebral Haemorrhage in China (2014)", all cases were examined by head CT at admission and were diagnosed as basal ganglia hemorrhage; (2) Patients with a hypertension history; (3) First cerebral haemorrhage; (4) Age younger than 75 years old; (5) The amount of bleeding in the basal ganglia was 15 ~ 30 ml. (6) The onset time is within 24 h. Exclusion criteria: (1) Patients with severe coagulation dysfunction or severe basic diseases; (2) Patients with surgical contraindications; (3) Bleeding entered into the ventricle; (4) Previous use of antiplatelet or anticoagulant medication, steroids, or immunosuppressants; (5) Prior onset of other neurological diseases, such as intracranial tumour, stroke, or severe head trauma; and (6) CTA examination of the head revealed cerebral aneurysm and cerebrovascular malformation. (7) Other systemic diseases, such as autoimmune disease, uraemia, cirrhosis, cancer, and chronic lung and heart diseases.

## Methods

General clinical data of patients were collected: sex, age, haematoma volume on admission, time from onset to visit, blood pressure on admission, Glasgow coma scale (GCS) score on admission and NIHSS score on admission. The NIHSS score and haematoma volume were evaluated on the 1st, 3rd, 7th and 30th days after admission, and the complications of the two groups were counted. Clinical management followed the guidelines for cerebral haemorrhage.

### Calculation of cerebral haematoma volume

According to the CT cross-section, the largest layer of high-density area was selected, The longest diameter (cm) and the widest diameter (cm) of haematoma were measured on the imaging system, and the slice thickness was read on the CT film. The number of slices was the total number of slices showing all haematomas. The amount of haematoma in the brain was calculated by using the multifield formula: haematoma volume (ml) = 1/2 × longest diameter (cm) × widest diameter (cm) × layer thickness (cm) × layers [8]. Two neurosurgery specialists read CT films without knowing the other information of the patient, and calculated the volume of the haematoma separately. When the difference between the calculated volumes of the two was greater than 5 ml, the third neurosurgeon calculated the volume. The same method was employed for neurological evaluation.

### Stereotactic minimally invasive drainage of intracranial haematoma

All patients in the experimental group received stereotactic minimally invasive drainage. Before the operation, patients were administered local anaesthesia with 2% lidocaine. Then, a Leskell-G stereotactic head frame was installed. Attention was given to the horizontal line of the head frame and the 15 degrees below the auditory canthal line. Then, 64-slice of Siemens head 3D CT scans were performed. Scanning parameters: kv/ma/rot.time, 120/270/1.0 s; scanning range, the scanning range covers the CT developing head frame; reconstruction matrix, 1024* 1024; fastest time resolution: 42 ms; spatial resolution: 241 p/cm; positioning line: listen to the angle line (OM); thickness of reconstruction layer: 1 mm: reconstruction interval: 1 mm; scanning method: 3D-Scan. Acquisition was performed 2 ~ 3 times to reduce human error. The layer with the largest haematoma area was selected as the stereotactic puncture layer, and the puncture target was generally selected 1/3 below the haematoma, establishing a rectangular coordinate system, and directly measuring and calculating the target coordinates (X, Y, Z spatial coordinates) in the CT 3D reading system. Then, according to the 3D CT image, the puncture path and angle were measured, and the surgical incision was determined. The preoperative target was measured and proofread by two neurosurgeons. After the target point was calculated, the patient returned to the operating room and was placed in the supine position. The Leskell headstock was connected and fixed with the special operating table for neurosurgery through an adapter. Routine ECG monitoring and disinfection were performed. The scalp was cut according to the incision calculated before the operation. The incision was generally 2 cm in size and the dura mater was cut approximately 5 mm after drilling the skull. Attention should be given to avoid excessive outflow of cerebrospinal fluid. The Leskell guide arc was installed, and the drainage tube was inserted under the guidance of the stereotactic instrument to reach the target point. During the operation, approximately 5 ml of haemorrhage was removed with 1 ml of negative pressure, and the same amount of normal saline was injected for replacement.Then, the operation was completed. The CT was reviewed after the operation. Fifty thousand units of urokinase were injected into the haematoma cavity to dissolve the haematoma, and the drainage tube was clamped for 3 h.The number of urokinase injections in the haematoma cavity was determined according to the drainage situation.

### Observation indicators

The general clinical data of the two groups were compared. The haematoma volume and NIHSS score were recorded on the 1st, 3rd, 7th and 30 days after treatment. The complications of the two groups after treatment were counted, including pulmonary infection, secondary bleeding, venous thrombosis of the lower limbs and stress ulcers.

### Statistical methods

SPSS 20.0 statistical software was used to analyse the collected data and the measurement data conforming to a normal distribution are expressed as the mean ± standard deviation ($\stackrel{-}{\mathrm{x}}$±s). The comparison between the two groups adopts was performed with independent sample T tests and variance analysis tests. Counting data were expressed by the number of cases and percentage (%). The X^2^ test or rank sum test was used according to the situation, and the ROC curve was used to calculate the Jordan index. The neurological score of the two groups was analysed, and the differences were statistically significant (*P* < 0.05).

## Results

### Comparison of general clinical data between the two groups

No significant differences in age, sex, onset time, haematoma volume, systolic blood pressure, diastolic blood pressure, GCS score and NIHSS score were noted between the two groups (*P* > 0.05, Table 1).

**Table 1:** Comparison of clinical data between two groups ($\stackrel{-}{\mathrm{x}}$±s)

	Conservative treatment group	Stereotactic group	Statistical values	*P*-value
	*n* = 30	*n* = 30		
Age (years)	60.23 ± 7.87	58.2 ± 8.40	0.967^a^	0.337
Gender (male, case,%)	43.33%	46.67%	0.067^b^	0.795
Time from onset to visit (hours)	3.77 ± 2.51	3.23 ± 2.25	0.866^a^	0.390
Haematoma volume at admission (ml)	20.96 ± 3.51	22.56 ± 3.25	-1.816^a^	0.074
Systolic blood pressure at admission (mmHg)	180.63 ± 12.78	185.03 ± 18.74	-1.062^a^	0.292
Diastolic Blood Pressure at Admission (mmHg)	99.97 ± 6.40	96.97 ± 6.53	1.797^a^	0.077
Evaluation (score) of GCS at admission	12.36 ± 1.69	12.27 ± 1.46	0.245^a^	0.807
Admission NIHSS score (points)	13.20 ± 3.54	12.77 ± 3.38	0.485^a^	0.629

Note: ^a^ is T value, and ^b^ is x^2^ value

### Comparison of haematoma volume and NIHSS score between the two groups

**Fig. 1:**
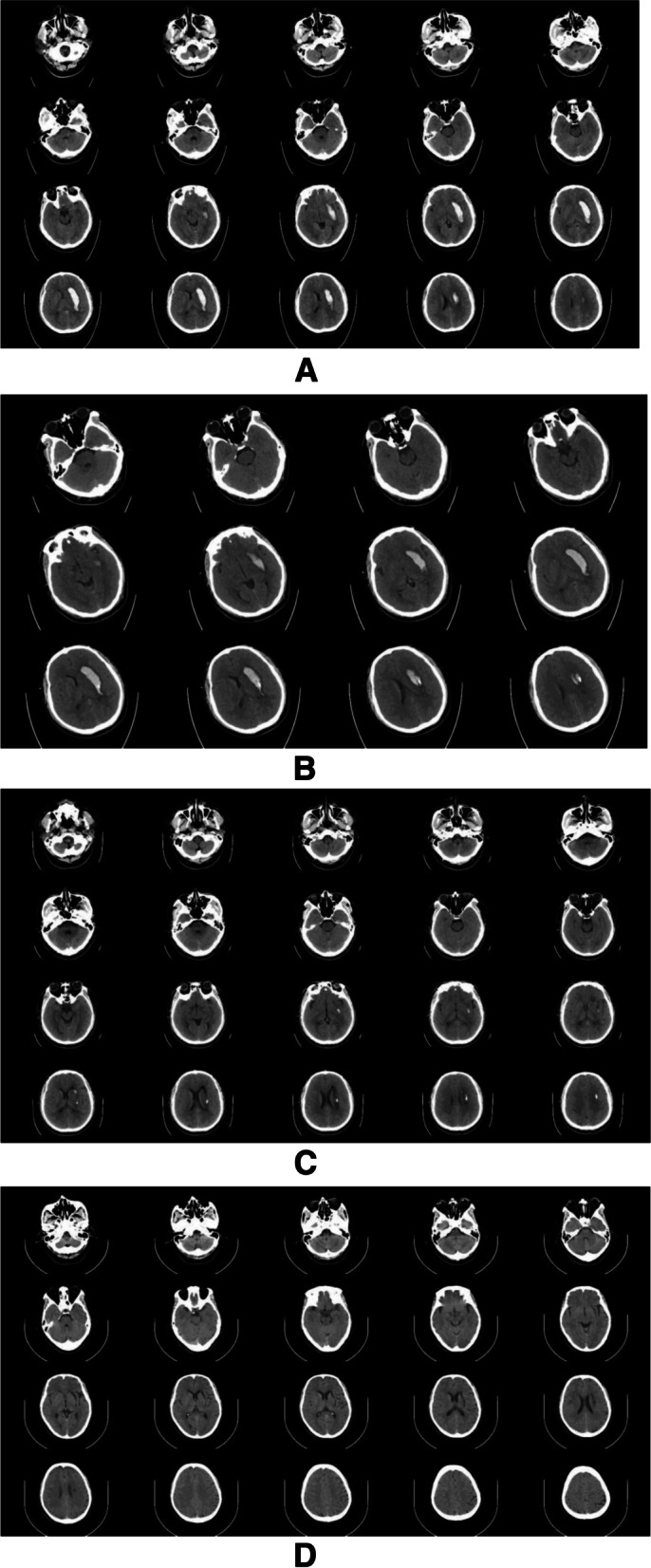
CT of patients with cerebral haemorrhage in the basal ganglia undergoing stereotactic catheterization and drainage. **A**. Preoperative head CT showed that the amount of bleeding was approximately 25 ml. **B**. The deformed position of three-dimensional CT on the first day after the operation indicates that the drainage tube is in a good position. **C**. On the third day after the operation, CT of the head showed that the haematoma was almost completely drained. **D**. On the 30th day after the operation, CT showed the soft focus after haematoma drainage

Patients in the two groups were followed up at 1, 3, 7 and 30 days after treatment, and the head CT was reexamined. The haematoma volume was calculated according to the Tada formula and the haematoma volume and NIHSS score were evaluated and recorded at different follow-up time points. (1) The change in haematoma volume (Table 2): the change in haematoma volume between the two groups was statistically significant (P < 0.01). In the stereotactic group, the haematoma was drained within 3 days (Fig. 1). The absorption time of haematoma in the conservative treatment group was approximately 1 month, which was significantly longer than that in the stereotactic drainage group. (2) NIHSS score (Table 3): One day after treatment, no significant difference was noted between the two groups (*P* > 0.05). However, significant differences in scores on the 3rd, 7th and 30th days after treatment were observed (*P* < 0.05), and the neurological deficit scores in the stereotactic group were significantly lower than those in the conservative treatment group.

**Table 2:** Haematoma volume at different time nodes in the two groups ($\stackrel{-}{\mathrm{x}}$±s, ml)

	Conservative treatment group	Stereotactic group	t value	*P* value
Treatment for 1 day	22.08 ± 4.26	17.70 ± 4.54	3.85	0.000
Treatment for 3 days	22.08 ± 4.26	5.03 ± 3.49	16.92	0.000
Treatment for 7 days	22.06 ± 4.08	2.46 ± 1.69	24.26	0.000
Treatment for 30 days	2.47 ± 2.75	0.00 ± 0.00	4.91	0.000

**Table 3:** NIHSS score at different time points of the two groups ($\stackrel{-}{\mathrm{x}}$±s, points)

	Conservative treatment group	Stereotactic group	t-value	*P*-value
Treatment for 1 day	13.10 ± 3.60	12.60 ± 3.19	0.569	0.572
Treatment for 3 days	13.96 ± 4.01	12.13 ± 2.92	2.023	0.048
Treatment for 7 days	15.10 ± 3.04	10.80 ± 1.90	6.563	0.000
Treatment for 30 days	6.76 ± 2.01	3.83 ± 1.20	6.850	0.000

### Effect of stereotactic surgery on neurological score of moderate and small amount of basal ganglia haemorrhage (Fig. 2)

**Fig. 2:**
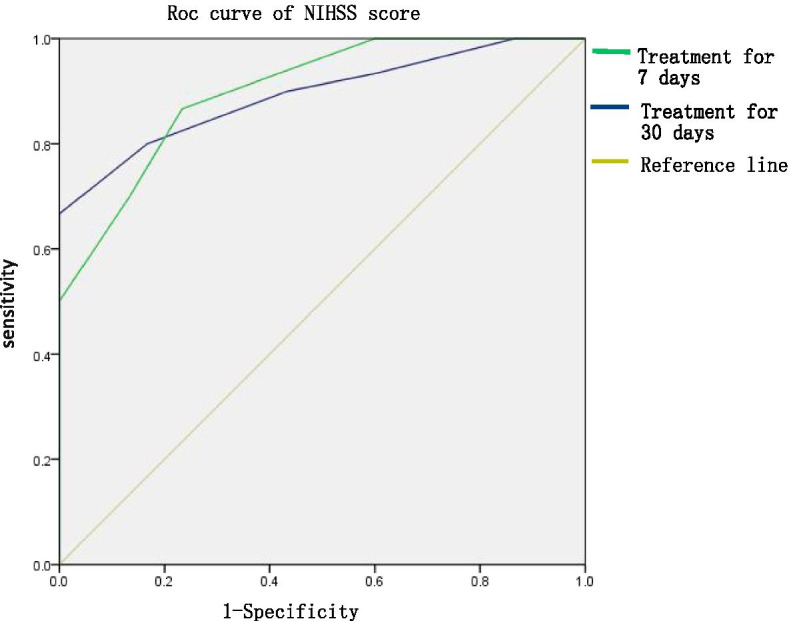
Effect of stereotactic surgery on the prognosis of moderate and small basal ganglia haemorrhages

ROC curve analysis revealed the sensitivity and specificity of stereotactic surgery on the neurological score of patients with small and medium basal ganglia haemorrhages. The sensitivity, specificity, Jordan index and area under the curve were 0.867, 0.767, 0.634 and 0.893, respectively, at 7 days after treatment (*p* = 0.000), respectively. The sensitivity, specificity, Jordan index and area under the curve of the NIHSS score were 0.800, 0.833, 0.633 and 0.901 (*P* = 0.000) at 30 days after treatment, respectively.

### Comparison of complications (Table 4)

**Table 4:** Complications after treatment in the two groups (*n* = 30, n%)

	Conservative treatment group	Stereotactic group	x^2^ value	*P* value
Lung infection	9 (30%)	2 (6.67%)	5.364	0.021
Secondary bleeding	1 (3.33%)	2 (6.67%)	0.345	0.557
Venous thrombosis of lower etremities	12 (40%)	5 (16.67%)	3.955	0.047
Stress ulcer	5 (16.77%)	6 (20%)	0.109	0.741

Compared with the conservative treatment group, the incidence of complications of pulmonary infection and venous thrombosis of the lower limbs in the stereotactic group was lower, and the difference was statistically significant (*P* < 0.05). No significant differences in complications of secondary bleeding and stress ulcers were noted between the two groups (*P* > 0.05).

## Discussion

Hypertensive cerebral haemorrhage is a common cerebrovascular disease in the clinic, and the most common bleeding site is the basal ganglia. The previous view was that conservative medical treatment should be employed when the bleeding volume is low (supratentorial < 30 ml, infratentorial < 10 ml) as this situation is not life-threatening. Studies have shown that if the haematoma is not cleared in a timely manner and effectively, it will exacerbate brain oedema and damage nerve function. Given that the patient's condition will deteriorate with time, the degree of nerve function damage will continue to increase, which is not conducive to patient rehabilitation [9, 10]. In the early stage, timely haematoma removal can alleviate secondary brain injury, effectively reduce intracranial pressure, improve cerebral perfusion, reduce complications and reduce the incidence of sequelae of cerebral haemorrhage [11, 12]. Therefore, increasing attention has been given to the surgical treatment of cerebral haemorrhage [13]. With the development of medical technology and the concept of microinvasion, the clinical application of minimally invasive surgery for cerebral haemorrhage, which significantly reduces the surgical trauma, is gradually increasing. Endoscopic surgery can remove the haematoma at an early stage and stop the bleeding. The application of stereotactic techniques in cerebral haemorrhage is based on the combination of medical technology and minimally invasive concepts [14]. Stereotactic haematoma puncture and drainage can reduce tissue damage, and has obvious advantages for deep haematomas, such as the brain stem, thalamus and basal ganglia. The results of this study are as follows.

### Effect of stereotactic surgery for small and medium basal ganglia haemorrhages

Brain injury caused by cerebral haemorrhage can be divided into primary brain injury and secondary brain injury. Primary brain injury refers to the direct trauma to local neurons caused by mechanical compression in the early stage of haemorrhage [15]. Secondary brain injury refers to cell damage and oedema caused by haemorrhage decomposition products and inflammatory reactions mediated by haemorrhage, which can aggravate neurological dysfunction [16]. Patients with moderate or small basal ganglia haemorrhages were hospitalized for a long time, and haematoma absorption typically occurred in approximately 3 ~ 4 weeks. With the arrival of the peak period of oedema, patients may experience deeper consciousness disorders, which lead to a significant increase in complications, such as pulmonary infection, venous thrombosis of the lower limbs and hemiplegia. Compared with conservative medical treatment, stereotactic surgery can better improve the prognosis of patients with small and medium cerebral haemorrhages [7]. This finding is consistent with our research. In this study, the evacuation speed of haematomas in the stereotactic surgery group is obviously faster than that in conservative treatment group. The method can alleviate the mechanical compression of the haematoma and reduce the damage of toxic substances released into neurons and glial cells during the decomposition and absorption of the haematoma, which is conducive to improving patients' consciousness disorders and reducing bleeding-related complications. In addition, this process facilitates early rehabilitation treatment; thus, the patients' GCS score and NIHSS scores can be improved [17].

### Indications of stereotactic surgery

Regarding indications of minimally invasive cerebral haemorrhage surgery, the Glasgow coma scale, operation time, haematoma volume and other aspects have detailed requirements. Given the need to install the head frame in stereotactic surgery, the installation of the head frame may cause secondary injury, blood pressure pulsation, or rebleeding. To ensure a good prognosis, it is necessary to strictly grasp the indications for surgery. Specifically, (1) The GCS score was 10 ~ 15 points, and the patient was a mild conscious disorder or conscious patient; (2) Check the head CT before installing the Leksell head frame. If the haematoma is obviously enlarged and the haematoma volume exceeds 30 ml, we may use endoscopic surgery or craniotomy to remove the haematoma. (3) The timing of the operation remains controversial. Surgical treatment of cerebral haemorrhage can be classified into ultra-early operation (within 6 h), early operation (6 h ~ 3 d) and delayed operation (over 3 d) [18].Studies have shown that among patients with hypertensive haematoma, the haematoma continues to enlarge in 36% of the patients within 3 h after the onset of the disease, and the haematoma continues to increase in 17% of the patients within 6 h. Scaggiante et al. report that for patients with cerebral haemorrhage, minimally invasive surgery seems to be beneficial in a 24- to 72-h window [19]. Wu et al. believe that intracranial haemorrhage in patients is still in the active stage within 6 h of onset [20]. Therefore, for safety considerations, they think that the interval between onset and surgery should be greater than 6 h. However, they did not compare the outcomes between patients who underwent surgery before and after 6 h. We believe that 6 to 24 h after cerebral haemorrhage is an ideal time window for minimally invasive treatment. However, whether this time period is the best treatment time window still requires further research. In our study, 2 patients had postoperative haematoma enlargement, but it did not cause the patient’s neurological function to deteriorate. After strengthening dehydration to reduce intracranial pressure and increasing number of injections of urokinase into the haematoma, the haematoma was completely drained and the patients passed the perioperative period safely. No patient experienced haematoma enlargement after urokinase injection.

### Advantages of stereotactic surgery

(1) Compared with conservative treatment, haematoma drainage is fast and thorough. By injecting urokinase into the haematoma cavity, most patients in this group experienced complete drainage in approximately 3 days. Minimally invasive drainage is conducive to early relief of haematoma compression, relieving brain oedema and reducing the damage of toxic substances released to neurons and glial cells during the decomposition and absorption of haematoma [21]. (2) Less trauma and accurate positioning. This methodology can accurately locate the puncture target and puncture path, and the puncture target is generally selected at 1/3 ~ 1/4 below the haematoma. We designed incision and puncture paths using a stereotactic surgery planning system before the operation, which can avoid functional areas and blood vessels and secondary injuries [22]. (3) The incision, target point and puncture path were planned before stereotactic surgery. In addition, the operation was simple and performed over a short period of time.

### Shortcomings and improvements

(1) Stereotactic surgery cannot solve and prevent the problem of intraoperative cerebral haemorrhage, so it is necessary to wait until the haemorrhage stabilizes. The drainage tube used was a silica gel drainage tube with an outer diameter of 4 mm, which was soft in texture. When puncturing, the tube is slowly rotated to the target point, and 1 ml of fluid is removed under negative pressure. The amount of fluid removed from the haematoma during the operation does not exceed 5 ml to avoid emptying the haematoma too quickly and reduce the risk of rebleeding to a certain extent. (2)A certain risk of infection after surgery exists. Because urokinase needs to be injected many times to dissolve the haematoma after the operation, the drainage tube will be placed for a long time, which will lead to an increased risk of infection. We strictly performed aseptic operation, injected urokinase through a medical three-way valve and removed drainage tubes within 5 days when possible to effectively reduce the risk of infection. (3) CT involves radiation, so a specific time interval must pass before reexamination using CT, which may result in untimely evaluation of the dynamic changes of haematoma in two groups. With the development of medical imaging, if the changes in haematoma can be dynamically monitored, the conclusion of this study may be more convincing.

## Conclusion

For patients with medium and small cerebral haemorrhages in the basal ganglia with neurological deficits, the aim of treatment should be to reduce the degree of neurological deficit, reduce complications and improve the quality of life of patients. Stereotactic drainage is a safe and effective surgical method that offers precise positioning, less trauma and quick haematoma drainage and can be used as the first choice for treatment. Because this is a single-center prospective study with a relatively small sample size and the follow-up time was short, the number of cases continues to increase. Thus, the sample size is expected in increase, and the follow-up time will be extended in further studies. A larger multicentre prospective study is needed to further confirm the above conclusion.

## Data Availability

All data are fully available without restriction.
